# Zinc Oxide Nanoparticles Influence Microflora in Ileal Digesta and Correlate Well with Blood Metabolites

**DOI:** 10.3389/fmicb.2017.00992

**Published:** 2017-06-02

**Authors:** Yanni Feng, Lingjiang Min, Weidong Zhang, Jing Liu, Zhumei Hou, Meiqiang Chu, Lan Li, Wei Shen, Yong Zhao, Hongfu Zhang

**Affiliations:** ^1^College of Animal Science and Technology, Qingdao Agricultural UniversityQingdao, China; ^2^Core Laboratories of Qingdao Agricultural UniversityQingdao, China; ^3^College of Marine Science and Technology, Qingdao Agricultural UniversityQingdao, China; ^4^State Key Laboratory of Animal Nutrition, Institute of Animal Sciences, Chinese Academy of Agricultural SciencesBeijing, China

**Keywords:** ZnO nanoparticles, microflora, ileal digesta, metabolism, correlation

## Abstract

Zinc oxide nanoparticles (ZnO NPs) are used widely in consumer and industrial products, however, their influence on gut microbiota and metabolism and their mutual interactions are not fully understood. In this study, the effects of ZnO NPs on ileal bacterial communities, plasma metabolites, and correlations between them were investigated. Hens were fed with different concentrations of ZnO NPs [based on Zn; 0 mg/kg (control), 25 mg/kg, 50 mg/kg, and 100 mg/kg] for 9 weeks. Subsequently, ileal digesta and blood plasma were collected for analysis of microflora and metabolites, respectively. The V3-V4 region of the 16S rRNA gene of ileal digesta microbiota was sequenced using the Illumina HiSeq 2500 platform. The predominant bacterial community in the ileum belongs to the phylum Firmicutes. The richness of the bacterial community was negatively correlated with increasing amounts of ZnO NPs (*r* = -0.636, *P* < 0.01); when ZnO NP levels were at 100 mg/kg, microbiota diversity was significantly decreased (*P* < 0.05). The community structure determined by LEfSe analysis indicated that Bacilli, Fusobacteria, and Proteobacteria were changed, and *Lactobacillus* was reduced by ZnO NPs. Moreover, metabolism as analyzed by nuclear magnetic resonance (NMR) indicated that glucose, some amino acids, and other metabolites were changed by ZnO NPs. Choline, lactate, and methionine were positively correlated with bacterial richness. In summary, ZnO NPs could influence the levels of microflora in ileal digesta, particularly *Lactobacillus*. Furthermore, the richness of the microbiota was related to changes in choline, lactate, and methionine metabolism.

## Introduction

Zinc oxide nanoparticles (ZnO NPs) are a new source of zinc with a diameter of <100 nm; they are widely used in consumer and industrial products. They offer excellent properties for antibacterial action, treatment of allergic diseases, and anti-cancer drug delivery (Kim et al., 2014; Patel et al., 2016). It is reported that ZnO NPs can effectively inhibit pathogenic yeast and *Candida albicans* (Lipovsky et al., 2011), with improved antibacterial action over copper oxide nanoparticles (CuO NPs) and ferric oxide nanoparticles (Fe_2_O_3_ NPs; Azam et al., 2012). Silva and Girard (2016) also discovered that ZnO NPs could prevent human eosinophil apoptosis through inhibiting caspase-1 and caspase-4. The adverse effects of ZnO NPs on organisms have been investigated under many conditions and the toxic effects are highly related to NP size and shape (Saptarshi et al., 2015). The augmentation of reactive oxygen species (ROS) and Zn^2+^ are the greatest potential causes of ZnO NP cytotoxicity (Wang et al., 2014). Furthermore, ZnO NPs have some influence on metabolism as evidenced by metabolites in mouse urine (Yan et al., 2012) and disturbed glucose metabolism in lung epithelial cells (Lai et al., 2015).

The bacterial community plays an important role in nutrition, immune system development, metabolic homeostasis, and anti-pathogenic action for humans and animals (Shin et al., 2011; Pan and Yu, 2014). Many factors can affect gut microflora, including the host, different diseases, diets, and additives, etc. It has been shown that fructose dianhydride-enriched caramel (FC) and the garlic derivative propyl propane thiosulfonate (PTS-O) influence the composition of ileal mucosa-associated microbiota (Ruiz et al., 2015), while phytogenic feed additives could increase *Lactobacillus* levels in the ceca of broiler chickens (Murugesan et al., 2015). Furthermore, the exogenous enzyme improves nutrient digestibility and reduces the number of *E. coli* (Rodriguez et al., 2012). Short-chain fatty acids and monoglycerides inhibit the contamination of *Campylobacter jejuni* in broiler chickens (Guyard-Nicodeme et al., 2016). Reed et al. (2015) proved that the richness and diversity of cecum microbiota were altered in chickens with chronic dietary Zn deficiency. Shao et al. (2014) found that zinc regulated the cecal microbial community by increasing the number of total bacteria and beneficial *Lactobacillus* bacteria, and reducing the number of *Salmonella* in broilers. The antibiotic zinc bacitracin induced specific changes in the cecal microbiota of broiler chicken (Gong et al., 2008; Torok et al., 2011; Costa et al., 2017; Crisol-Martinez et al., 2017). ZnO NPs produced attractive antibacterial properties because of the increased specific surface area as the reduced particle size with enhanced particle surface reactivity (Sirelkhatim et al., 2015). Hu et al. (2017) has found that the microbial species richness in the EBPR system was reduced by ZnO NPs and higher concentration ZnO NPs induced more microbial community shift. ZnO NPs-caused antibacterial effects may be due to a combination of Zn^2+^ ions and induction of ROS formation (Frohlich and Frohlich, 2016).

Although there are still few reports on the possible toxicological effects of nanoparticles on microbiota/microbiome, and on their possible clinical consequences, available data suggest that carbon nanotubes (CNTs), TiO2 NPs, CeO2 NPs, ZnO NPs, SiO_2_ NPs, and Ag NPs may affect the microbiota in intestine (Pietroiusti et al., 2016). Ag NPs and Ti_2_O NPs caused changes in the microbiota and mucus in intestine (Mercier-Bonin et al., 2016). Taylor et al. (2015) used an *in vitro* colon model found that ZnO NPs caused significant changes to the microbial community’s phenotype, which might be related to overall health effects. Moreover, it has been shown that ZnO NPs can affect soil microflora and the growth of dominant bacteria, namely *Bacillus subtilis* and *Pseudomonas aeruginosa* (Ge et al., 2011; Santimano and Kowshik, 2013). However, the effects of ZnO NPs on gut microflora of domestic animals and on the relationship between metabolites and gut microflora are not yet understood. Since gut microflora are of great importance to metabolism, the objective of this investigation was to explore the influence of ZnO NPs on the bacterial community of hen ileal digesta, the metabolites in the plasma, and the relation between them.

## Materials and Methods

### The Characterization of ZnO Nanoparticles

Zinc oxide nanoparticles were synthesized by Beijing DK Nano Technology Co. LTD (Beijing, China) as reported in our recent publications (**Supplementary Figure S1**) (Zhao et al., 2016). The characteristics of ZnO NPs (morphology, size, agglomeration, etc.) were determined by transmission electron microscopy (TEM; JEM-2100F, JEOL Inc., Japan) and dynamic light scattering (DLS) particle size analyzer (Nano-Zetasizer-HT, Malvern Instruments, United Kingdom).

### Animal Husbandry and Sample Collection

All animal experimental procedures were followed according to the regulations of the animal ethics committee of the Qingdao Agricultural University. All Jinghong-1 hens used in the experiment were housed in a commercial poultry building at Maochangyuan Co. (Qiangdao, China). Diets and nutrients are shown in Supplementary Table S1. Briefly, 160 six-week-old hens were equally divided into four different treatments: control (C), no zinc addition; ZnO-NP-25 (25 mg/kg ZnO NPs; based on the concentration of zinc); ZnO-NP-50 (50 mg/kg ZnO NPs); and ZnO-NP-100 (100 mg/kg ZnO NPs) with four repeats in each treatment (10 hens/repeat). At 15 weeks, two hens from each repeat were euthanized after recording body weight and collection of blood samples. Ileal digesta (eight samples of each group) was packaged and immersed in liquid nitrogen before being stored at -80°C for DNA isolation. Blood samples (eight samples of each group) were centrifuged at 3000 rpm for 15 min at 4°C and the plasma was stored at -80°C for metabolite detection.

### The Sequencing of Microbiota from Ileal Digesta Samples

#### DNA Extraction

Total genomic DNA of ileal digesta was extracted using an E.Z.N.A.^®^ Stool DNA Kit (Omega Bio-tek Inc., United States) according to the manufacturer’s introduction. DNA quantity and quality were monitored with NanoDrop 2000 (Thermo Scientific, United States) and 1% agarose gel. Totally 32 ileal digesta DNA samples were extracted and the two ileal digesta DNA from the same replicate were equally mixed and 16 samples were applied for sequencing (*n* = 16).

#### Library Preparation and Sequencing

The V3-V4 region of the 16S rRNA gene was amplified using the primers MPRK341F (5′-CCTAYGGGRBGCASCAG-3′) and MPRK806R (5′-GGACTACNNGGGTATC TAAT-3′) (Yu et al., 2005) with Barcode. The volume of PCR reactions was 30 μL with 15 μL Phusion^®^ High-Fidelity PCR Master Mix (New England Biolabs), 0.2 Mm primers and 10 ng DNA. The thermal cycle was carried out with initial denaturation at h, followed by 30 cycles of 98°C for 10 s, 50°C for 30 s and 72°C for 30 s, and a final extension at 72°C for 5 min. PCR products were mixed in equidensity ratios and purified using a GeneJET Gel Extraction Kit (Thermo Scientific, United States). The sequencing libraries were generated with NEB Next^®^ Ultra^TM^ DNA Library Prep Kit for Illumina (NEB, United States) following the manufacturer’s recommendations and index codes were added. Lastly, the library was sequenced on the Illumina HiSeq 2500 platform and 250 bp paired-end reads were generated at the Novo gene. The paired-end reads were merged by using FLASH (V1.2.7^1^). The quality of the Tags was controlled in QIIME (V1.7.0^2^) as according to the protocol described by Caporaso et al., 2010), meanwhile all chimeras were removed (Edgar et al., 2011; Haas et al., 2011). The “Core Set” of the Greengenes database^3^ was applied for classification, and sequences with ≥97% similarity were assigned to the same operational taxonomic units (OTUs) (Magoc and Salzberg, 2011).

#### Analysis of Sequencing Data

Operational taxonomic unit abundance information was normalized using a standard of sequence number corresponding to the sample with the least sequences. The alpha diversity index was calculated with QIIME (Version 1.7.0). The Unifrac distance was obtained by QIIME (Version 1.7.0), and PCoA (principal coordinate analysis) was performed using R software (Version 2.15.3). The Linear discriminate analysis effect size (LEfSe) was performed to determine differences in abundance, the threshold of LDA score was 4.0. GraphPad Prism7 software was used to produce the graphs.

### Analysis of Metabolites in Blood Plasma

#### Blood Plasma Preparation and Nuclear Magnetic Resonance (NMR) Spectrum Processing

The detection of metabolites in hen plasma followed the method reported by Wan et al. (2016). In total, 600 μL of solution (including 200 μL plasma, 80 μL D_2_O and NaCl media to a final concentration of 0.9% NaCl) was transferred to a 5 mm nuclear magnetic resonance (NMR) tube. A Bruker Avance III 600 MHz NMR spectrometer equipped with an inverse detection cryoprobe (Bruker Biospin, Rheinstetten, Germany) was used to obtain the ^1^H NMR spectra for plasma samples.

#### Data Analysis of the ^1^H NMR Spectra

The plasma spectra were corrected with phase and baseline distortions, the spectra were then referenced to TSPd_4_ (δ 0.000) and plasma (δ 0.5–8.5). Imperfect water suppression (δ 4.40–5.20) was excluded. The obtained spectra were normalized to the total sum of intensity and the data were analyzed with SIMCA-P software (version 11.0, Umetrics, Umea, Sweden). All Tukey’s multiple comparison tests using one-way ANOVA were performed with GraphPad Prism7. Correlation analysis was performed using SPSS version 22.

## Results

### Effects on Body Weight

**FIGURE 1:**
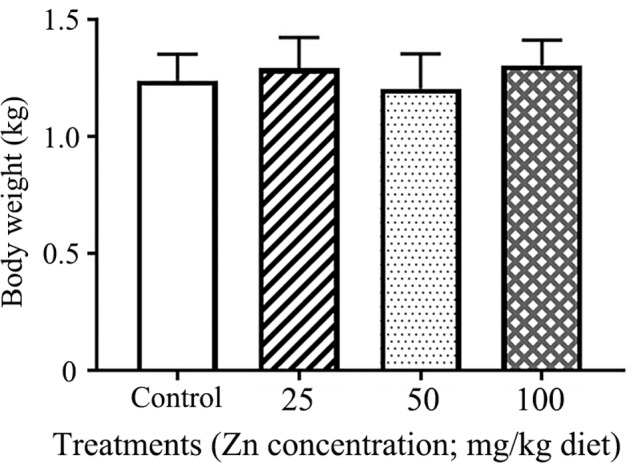
Effects of ZnO NP treatment on hen body weight. The *Y*-axis represents body weight (kg), and the *X*-axis represents the treatment group.

After 9 weeks of treatment, individual hen body weights were 0.92–1.48 kg, and these remained unaltered by treatments using different concentrations of ZnO NP supplement (**Figure 1**).

### Changes of Microflora in Ileal Digesta

#### Patterns of Predominant Bacterial Communities in Ileal Digesta

**FIGURE 2:**
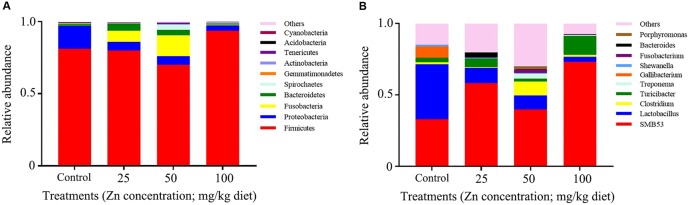
The relative richness of microbiota at phylum and genus level. The representative sequences for each OUT was used to annotate taxonomic information: **(A)** phylum, **(B)** genus.

Based on relative abundance, ZnO NPs altered the predominant bacteria in hen ileal digesta. In the control group, the predominant bacteria were Firmicutes, Clostridia and Bacilli, Clostridiale and Lactobacillales, *Clostridiaceae* and *Lactobacillaceae*, *SMB53*, and *Lactobacillus*; while Firmicutes, Clostridia, Clostridiale, *Clostridiaceae*, and *SMB53* were the predominant bacteria in ZnO NP treatments (**Figure 2** and **Table 1**).

**Table 1:** The relative abundance of gut microflora in ileal digesta (%).

Taxonomy	Phylum	Class	Order	Family	Genus
Control	Firmicutes 81.14	Clostridia 37.22	Clostridiales 37.22	Clostridiaceae 34.83	*SMB53* 33.03
	Proteobacteria 15.94	Bacilli 43.71	Lactobacillales 39.94	Lactobacillaceae 38.34	*Lactobacillus* 38.33
			Pasteurellales 7.96	Pasteurellaceae 7.84	*Gallibacterium* 7.81
			Others 5.47	Others 10.63	Others 14.89
ZnO25	Firmicutes 79.89	Clostridia 61.83	Clostridiales 61.83	Clostridiaceae 58.87	*SMB53* 58.18
	Proteobacteria 5.97	Bacilli 17.88	Lactobacillales 11.53	Lactobacillaceae 10.83	*Lactobacillus* 10.83
	Fusobacteria 7.70	Fusobacteriia 7.70	Fusobacteriales 7.70	Fusobacteriaceae 7.68	*Turicibacter* 6.00
	Bacteroidetes 5.03	Bacteroidia 4.98	Bacteroidales 4.98	Turicibacteraceae 6.00	*Bacteroides* 3.53
			Turicibacterales 6.00	Others 12.01	Others 20.20
ZnO50	Firmicutes 70.07	Clostridia 57.54	Clostridiales 57.54	Clostridiaceae 53.39	*SMB53* 39.94
	Proteobacteria 5.91	Bacilli 12.51	Lactobacillales 10.27	Lactobacillaceae 9.59	*Lactobacillus* 9.59
	Fusobacteria 14.39	Fusobacteriia 14.39	Fusobacteriales 14.39	Fusobacteriaceae 15.52	*Clostridium* 9.79
	Bacteroidetes 3.94	Bacteroidia 3.91	Bacteroidales 3.91	Spirochaetaceae 3.49	Others 30.05
	Spirochaetes 3.51	Spirochaetes 3.51	Spirochaetales 3.49	Others 12.89	
			Others 4.57		
ZnO100	Firmicutes 93.7092	Clostridia 75.43	Clostridiales 75.36	Clostridiaceae 74.59	SMB53 73.21
	Proteobacteria 3.81	Bacilli 18.18	Lactobacillales 4.44	Lactobacillaceae 3.62	*Lactobacillus* 3.57
			Turicibacterales 13.35	Turicibacteraceae 13.35	*Turicibacter* 13.35
			Others 3.66	Others 6.00	Others 7.38


*This table only documented the group unites taxa whose abundance (each) exceed 3.5% of the total.*

#### Diversity of Bacterial Communities in Ileal Digesta

**FIGURE 3:**
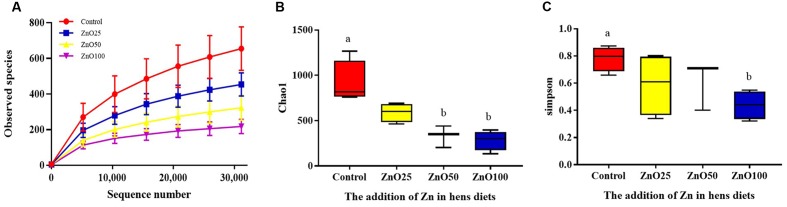
The richness and the diversity of the bacterial community. The alpha index of the ileal microbiota: **(A)** Rarefaction curve, **(B)** Chao1 index, **(C)** Simpson index.

**FIGURE 4:**
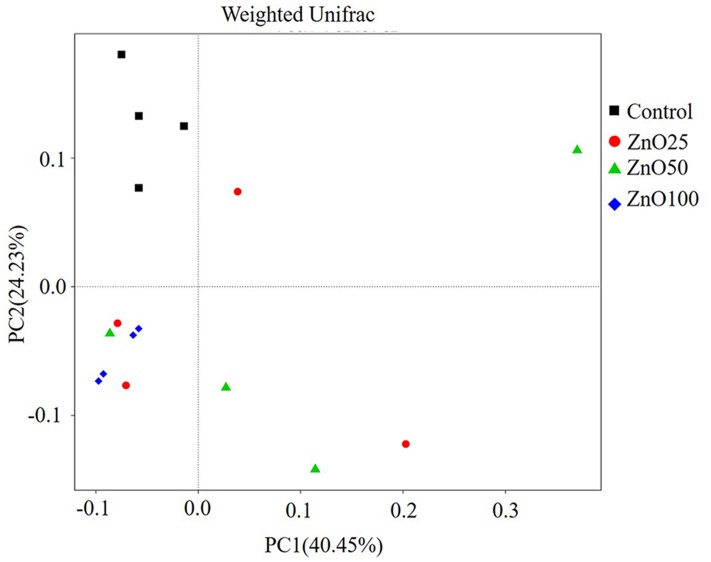
The PCoA of the microflora in different treatments. After sequencing, Unifrac distances were calculated by QIIME (Version 1.7.0) and the PCoA was performed with R software (Version 2.15.3).

In total, 815 828 clean tags were obtained, with an average efficiency of 83.37%. The rarefaction curve revealed that the sequence number was reliable for further analysis (**Figure 3A**), meanwhile, the observed species of ileal digesta microbiota were negatively correlated with increased levels of ZnO NPs (*R* = -0.636, *P* < 0.01, *n* = 16). Based on the alpha diversity index, richness (Chao1) and diversity (Simpson) were identified. Compared with the control group, richness was significantly decreased in the ZnO-NP-50 and ZnO-NP-100 treatments (**Figure 3B**), however, diversity was decreased only in treatment ZnO-NP-100 (**Figure 3C**). Moreover, based on the weighted unifrac distance, the PCoA visually showed that all ZnO NPs treatments were spatially separated from the control group, and Zn100 was the most distinctively different because of the low variability, and the explanation of PC1 and PC2 was >60% (**Figure 4**).

#### Characterization of Bacteria in Ileal Diagesta

**FIGURE 5:**
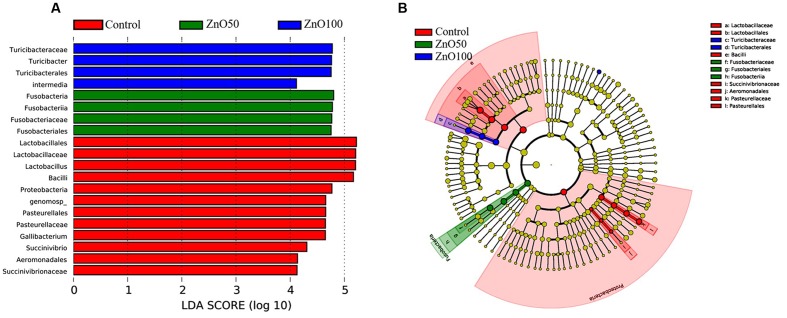
Differences of bacterial abundance: **(A)** LDA distribution, **(B)** Cladogram. Linear discriminate analysis effect size (LEfSe) was performed to determine the difference in abundance; the threshold of LDA score was 4.0.

To evaluate the difference in bacterial content between the four groups, LEFSe were performed with an LDA value of four. There were 12 taxa that were significantly different in the control group, ZnO-NP-50, and ZnO-NP-100. When compared with ZnO NP treatments, the abundance of Lactobacillales, Aeromonadales, and Pasteurellales were higher, and the Fusobacteriales and Turicibacterales were lower in the control group (**Figure 5**).

### Changes of Metabolites in Different Treatments

**FIGURE 6:**
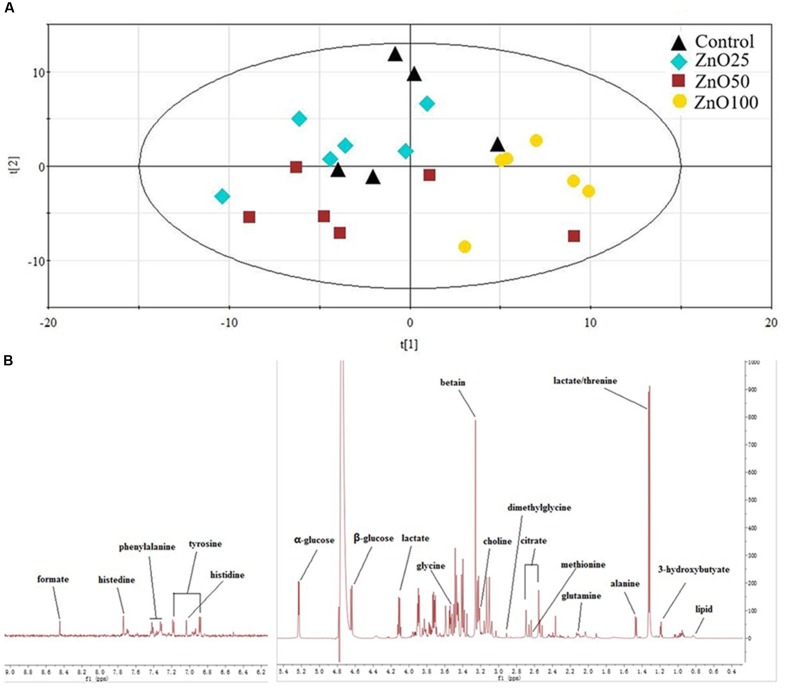
Metabolites in blood plasma: **(A)** the PCA of metabolites, **(B)** Typical 600 MHz ^1^H NMR spectra of plasma.

**FIGURE 7:**
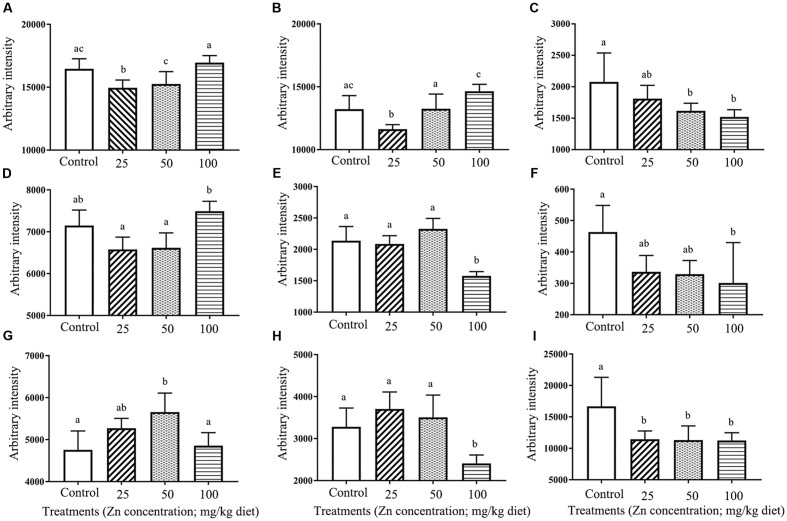
Change in metabolites under different treatments: **(A)** α-glucose, **(B)** β-glucose, **(C)** Choline, **(D)** Glycine, **(E)** Methionine, **(F)** Tyrosine, **(G)** Glutamine, **(H)** Citrate, **(I)** Lactate.

Lipids, glucose, choline, lactate, citrate, and amino acids were identified from the ^1^H NMR spectra. Data distribution was exhibit by PCA, and indicated that the ZnO-NP-100 treatment deviated from other groups (**Figure 6**). Metabolite analysis showed that levels of glucose, choline, lactate, citrate, glutamine, glycine, methionine, and tyrosine were altered by ZnO NP administration (**Figure 7**).

### Correlations between Bacterial Richness and Metabolites in Plasma

Since gut bacteria play a key role in food digestion, correlations between bacterial richness and metabolites were analyzed. The results showed that ileal digesta bacteria were positively related to choline, lactate, and methionine (**Table 2**).

**Table 2:** Correlations between the observed species and the major metabolites.

		Choline	Lactate	α-glucose	β-glucose	Glutamine	Lipid	Tyrosine	Glycine	Methionine	Citrate
Observed species	Pearson correlation	0.769^∗∗^	0.509^∗^	0.056	-0.190	-0.086	-0.385	0.304	0.243	0.590^∗^	0.229
	Significance (2-tailed)	0.001	0.044	0.836	0.481	0.753	0.141	0.252	0.364	0.034	0.393
	*N*	16	16	16	16	16	16	16	16	13	16


*^∗^*p* < 0.05; ^∗∗^*p* < 0.01.*

## Discussion

Zinc oxide nanoparticles under 100 nm in diameter have special bioactivity and are widely used in many aspects of our lives. Since their first use, the adverse effects of ZnO NPs have been of great concern and have been widely studied in cells, animals, and even human subjects (Taccola et al., 2011; Gao et al., 2016; Han et al., 2016). However, the effects of ZnO NPs on gut microflora and metabolites in domestic animals had not been studied to-date. In this investigation, pullets were used to investigate the effects of ∼30 nm ZnO NPs on the gut microflora and blood metabolites. The dose dependent influence of ZnO NPs was investigated by different dose of Zinc addition around 45 ppm (the recommend zinc dose for pullet).

### ZnO NPs Show Varying Effects on the Body Weight of Different Animal Species

Fathi et al. (2016) first added different amounts of 35–45 nm ZnO NPs (10 mg/kg, 20 mg/kg, and 40 mg/kg) to diets and noted that this could improve the bodyweight of broiler chickens. Results in the current research revealed that the body weight of young hens remained unchanged by the addition of ZnO NPs, which indicates that different animal species and different animal life spans might respond differently to ZnO NPs. Previously, we discovered that ZnO NPs could decrease yolk lipids content (Zhao et al., 2016). Moreover, as gut microflora are involved in the metabolism (Nicholson et al., 2012), the pullet were selected as a model to verify the correlations between gut microflora and metabolites in this study.

### ZnO NP Effect on Gut Microbiota Varied According to Concentration, Particle Size, and Animal Species

It is well known that ZnO NPs are good candidates for antimicrobial treatments for both G^+^ and G^-^ bacteria (Azam et al., 2012). They effectively inhibit pathogens such as *Staphylococcus aureus* (Geilich and Webster, 2013), *Campylobacter jejuni* (Xie et al., 2011), *Escherichia coli O157:H7* (Liu et al., 2009), *Listeria monocytogenes*, and *Salmonella Enteritidis* (Jin et al., 2009). To date, the influence of ZnO NPs on the ecology of bacteria in domestic animals has been little understood. In the current study, we assessed changes in bacterial communities found within the ileal digesta of young hens, and suggest that the administration of ZnO NPs can decrease bacterial richness. Furthermore, the influence was enhanced by increased ZnO NP concentration, and at the treatment level of 100 mg/kg it rearranged the predominant bacteria in ileal digesta.

The dominant bacteria in the intestines of animals and humans vary with organ, animal species and age. We found that Firmicutes (>70%) and Proteobacteria (>5%) were the dominant phyla of bacteria in the ileal digesta of all groups of young hens, and that ZnO NPs did not alter the relative abundance of Proteobacteria (Yausheva et al., 2016). In attempting to explain this disparity, firstly, *Eisenia fetida* are annelids, which are quite different to domestic animals. Secondly, bacteria throughout the entire intestinal tract were used for the latter authors’ experiment, while we only focused on bacteria in ileal digesta. Finally, the size and amount of ZnO NPs used were different; the above-mentioned authors used 100 nm and 1000 mg/kg, respectively, while 30 nm and 25, 50, and 100 mg/kg of ZnO NPs, respectively, were used in our investigation.

Reports have shown that *Lactobacillus acidophilus* (PTCC 1643) is inhibited by 1% ZnO NPs *in vitro* (Kasraei et al., 2014). This is of concern because *Lactobacillus* is the predominant bacteria in animal and human ilea (Torok et al., 2011; Turroni et al., 2014; Jiao et al., 2016). In the current study, we also noted that the number and relative abundance of *Lactobacillus* in ileal digesta was remarkably reduced by ZnO NPs. The relative abundance of *Lactobacillus* in the control group was about 38.33%; this agreed well with other studies and proved that *Lactobacillus* was the dominant bacteria in the ileum of young hens. As the dominant bacteria, it can be easily changed. It has been reported that *Lactobacillus*, especially *Lactobacillus aviaries*, is negatively correlated with the pathogen *Clostridium perfringens* in the ileum of broilers (Feng et al., 2010). Other authors found that a *Lactobacillus* population may be reduced through the feeding of antibiotic growth promoters (Lin et al., 2013). *Lactobacillus aviaries* and *Lactobacillus salivarius* are the predominant bacteria in the upper gastrointestinal tract of broilers (Gong et al., 2007). It has been confirmed that *L. salivarius* impedes broiler chicken performance by deconjugating bile salts (Harrow et al., 2007). On the other hand, our treatments produced no change in body weight compared with the control group. In our previous study, effects on egg laying frequency indicated that ZnO NPs decreased layer performance during the early period, while no difference was observed after 23 weeks of treatment (Zhao et al., 2016). It is therefore probable that *L. salivarius* is not sensitive to ZnO NPs.

*Turicibacter* is another dominant bacteria in the small intestine (Kamada et al., 2013), and its level could be increased by feeding an iron sulfate free diet (Werner et al., 2011). In the current study, ZnO NPs increased the relative abundance of *Turicibacter* in the 100 mg/kg ZnO NPs treatment. Interestingly, when compared with the control group, the number of *SMB53* was quite similar, while the relative abundance was increased in ZnO NPs treatments. The mechanism by which ZnO NPs affected *Turicibacter* and *SMB53* was unclear. *Turicibacter* is considered as harmful bacteria and it was increased by 100 mg/kg ZnO NPs treatment. This might suggest that bacterial tolerance has developed, or antibacterial activity has priorities in the complex environment.

Briefly, ZnO NPs affect the bacterial communities of ileal digesta. However, the research and reports on this subject were quite limited. Determination of the key mechanism (zinc, nanoparticles or both of them) involved in the changing the microbiota require further study.

### Metabolites in Blood Plasma Were Changed by ZnO NPs

Due to their small size, ZnO NPs are able to enter cells and influence cell functions, including metabolism. Lai et al. (2015) discovered that ZnO NPs (mean diameter 63.1 nm) located in lung cell mitochondria cause mitochondrial dysfunction and are a major source of ROS; in turn, the level of ROS affects energy metabolism. They also found that glucose consumption and lactate production were reduced at the concentration of 10 μg/ml. Yan et al. (2012) found that rats given 50 nm ZnO NPs (100, 300, and 1000 mg/kg) developed nephrotoxicity; the NPs changed kidney metabolism with a resulting increase in lactate and alpha-glucose and reduction in lipids and citrate in urine. The results of our current research did not all agree with earlier studies. We investigated blood plasma metabolites and the results showed that low levels of ZnO NPs (25 mg/kg) decreased glucose, while glucose (alpha and beta) was increased with increasing concentration of ZnO NPs and the level of beta-glucose was even higher than in the control. It is promising that ZnO NPs could decrease blood glucose at certain concentrations. Furthermore, we found that citrate was decreased at 100 mg/kg ZnO NPs; however, lactate and choline were statistically significantly reduced by ZnO NPs, and lipids were also changed in our study; these results did not agree; with the findings of Yan et al. (2012). Basically, the animals and the samples used in the studies were different. Furthermore the above mentioned authors examined metabolism following the addition of 1000 mg/kg ZnO NPs, that was 10-fold greater than our highest dosage. Although some differences were observed, it is certain that ZnO NPs can change metabolites by altering cell function.

### Metabolites Were Correlated with the Observed Species of Gut Microflora

Gut microbiota are associated with development, disease, and metabolism. They are involved in the metabolism of short-chain fatty acids (SFCAs), glucose, lipids, vitamins, and choline etc., which are essential substrates for body maintenance and production (Nicholson et al., 2012). In the current research, the PCoA on bacteria template and the PCA on plasma showed that the 100 mg/kg ZnO NPs treatment had significantly different results to the control group; this strongly suggests a connection between the gut bacterial community and metabolism. Nicholson et al. (2012) also reported that *Lactobacillus* is involved in the metabolism of bile acids, lipids, glucose, and D-lactate etc. We observed that bacterial numbers in ileal digesta were positively correlated with choline, lactate, and methionine, while other metabolites were not.

In summary, ZnO NPs are widely used, could decrease the richness of bacterial communities, and inhibit *Lactobacillus* content. Bacterial richness in the ileum was correlated with some metabolites, including choline, lactate, and methionine. Together, ZnO NPs might regulate metabolism directly in host cells and indirectly via changing gut microbiota.

## Data Access

The raw sequencing data generated in this study has been uploaded to the NCBI SRA database with the accession number: PRJNA361394 (http://www.ncbi.nlm.nih.gov/bioproject/PRJNA361394).

## Author Contributions

YF, LM, and YZ designed and wrote the manuscript. WZ, JL, HZ, and MC performed the experiments. ZH, LL, and WS provided the data analysis and paper writing.

## Conflict of Interest Statement

The authors declare that the research was conducted in the absence of any commercial or financial relationships that could be construed as a potential conflict of interest.

## Footnotes

1 http://ccb.jhu.edu/software/FLASH/

2 http://qiime.org/index.html

3 http://greengenes.lbl.gov/cgi-bin/nph-index.cgi

## Supplementary Material

**FIGURE S1 |** Characteristics of ZnO NPs. **(A)** Photo of ZnO NPs. **(B)** TEM image of ZnO NPs. **(C)** TEM photo of ZnO NPs in ovary indicated by the white arrow. **(D)** EDS picture of ZnO NPs in ovary, where three Zn peaks have shown.
