# Supplementary figures and images for: Zinc Oxide Nanoparticles Influence Microflora in Ileal Digesta and Correlate Well with Blood Metabolites
Source: Front Microbiol. 2017 Jun 2;8:992. doi: 10.3389/fmicb.2017.00992 (PMC5454036; doi:10.3389/fmicb.2017.00992)

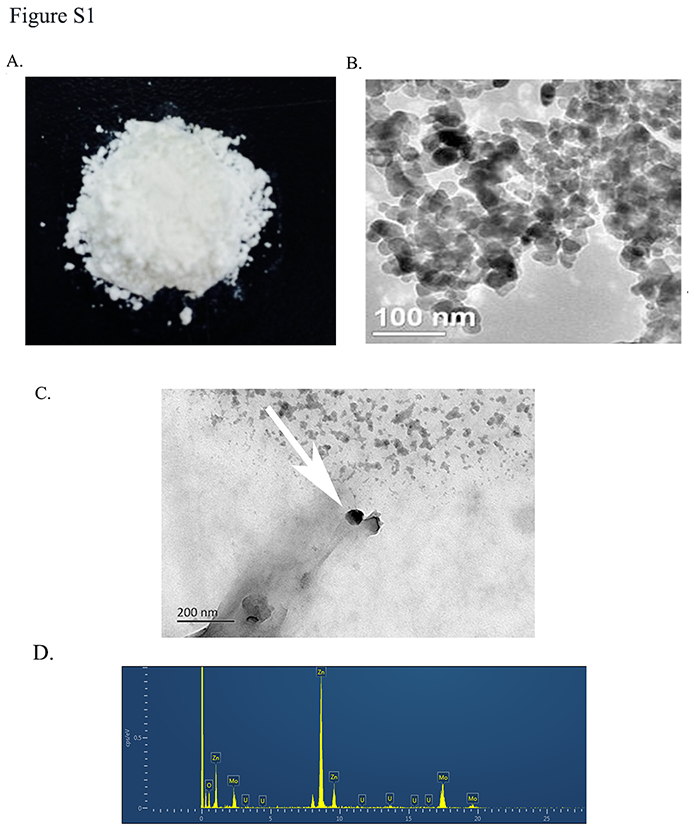

Supplement: Supplementary file 1 [file Image_1.tif]
